# Cyclotron resonance in HgTe/CdTe-based heterostructures in high magnetic fields

**DOI:** 10.1186/1556-276X-7-534

**Published:** 2012-09-26

**Authors:** Maxim S Zholudev, Anton V Ikonnikov, Frederic Teppe, Milan Orlita, Kirill V Maremyanin, Kirill E Spirin, Vladimir I Gavrilenko, Wojciech Knap, Sergey A Dvoretskiy, Nikolay N Mihailov

**Affiliations:** 1 Institute for Physics of Microstructures of the Russian Academy of Sciences, GSP-105, Nizhny Novgorod, 603950, Russia; 2 Laboratoire Charles Coulomb (LCC), Universite Montpellier II, Montpellier, 34095, France; 3 Laboratoire National des Champs Magnetiques Intenses (LNCMI-G), 25 rue des Martyrs, B.P. 166, Grenoble, 38042, France; 4 Institute of Semiconductor Physics, Siberian Branch of the Russian Academy of Sciences, pr. Lavrentieva 13, Novosibirsk, 630090, Russia

**Keywords:** Cyclotron resonance, HgTe/CdTe heterostructures, HgTe quantum wells, Far-IR magnetospectroscopy

## Abstract

Cyclotron resonance study of HgTe/CdTe-based quantum wells with both inverted and normal band structures in quantizing magnetic fields was performed. In semimetallic HgTe quantum wells with inverted band structure, a hole cyclotron resonance line was observed for the first time. In the samples with normal band structure, interband transitions were observed with wide line width due to quantum well width fluctuations. In all samples, impurity-related magnetoabsorption lines were revealed. The obtained results were interpreted within the Kane 8·8 model, the valence band offset of CdTe and HgTe, and the Kane parameter *E*_*P*_ being adjusted.

## Background

HgTe/CdTe-based quantum wells (QWs) exhibit a number of remarkable properties. At the critical HgTe QW thickness (6.3 to 7 nm depending on Cd content in the barrier), the forbidden gap is absent and both electrons and holes are characterized by the linear energy-momentum law of massless Dirac fermions [1,2]. When HgTe QW width exceeds this critical value, the energy band structure is inverted (the conduction band states are formed by p-type wavefunctions while s-type wavefunctions form the valence band states; see, e.g., [1,3] and references therein). In the inverted band structure regime, HgTe QWs are shown to be two-dimensional (2D) topological insulators that have attracted a great fundamental interest [1,2,4,5]. It was demonstrated [4] that a quantum spin Hall insulator state exists in such systems that can be destroyed by magnetic field due to crossing of Landau levels of different bands [6]. Actually, these two levels have recently shown to display the effect of the avoided crossing [7,8]. Hole-like symmetry of conduction-band Bloch functions enhances spin-dependent effects like the Rashba splitting that has been shown to achieve 30 meV [3,9]. Wide HgTe/CdTe QWs have an indirect band structure [10]. If the well is wide (above 12.5 nm), the side maxima of the valence band overlap with the conduction band. Then, the Fermi level can cross both valence and conduction bands and a semimetallic state can be implemented which has been revealed by magnetotransport measurements [11,12]. On the other hand, narrow HgTe QWs have been proposed as a material for detectors of THz radiation since they possess certain advantages over bulk HgCdTe solid solutions that are widely used for mid-infrared (IR) photodetectors. An alternative way to tune the QW structure is to admix Cd into a wide HgTe QW. In [13,14], 30-nm-wide Hg_1-*x*_Cd_*x*_Te QWs with a Cd content *x* > 0.13 are shown to have normal band structure. However, properties of such wells are not identical to those of normal-band-structure HgTe QWs with the same bandgap, namely wide HgCdTe QWs demonstrate indirect band structure, i.e., the side maximum in the valence band exceeds that in the center of the Brillouin zone. An informative method to probe the energy band structure both in bulk semiconductors and in QWs is the cyclotron resonance (CR) technique. However, at the moment, there have been no systematic studies on CR in HgTe/CdTe QWs with different band structures (*cf.*[6-9,13-19]). In this work, we present the first results on CR measurements in a semimetallic sample with wide HgTe QW (inverted band structure) as well as in two samples with normal band structures: narrow HgTe QW (for the first time) and wide HgCdTe (about 15% of cadmium).

## Methods

### Experimental

The structures under investigation were grown by molecular beam epitaxy on semi-insulating GaAs(013) substrates [20]. The ZnTe and thick relaxed CdTe buffer layers, a 100-nm Cd_*y*_Hg_1-*y*_Te lower barrier layer, a Hg_1-*x*_Cd_*x*_Te QW, and a 100-nm Cd_*y*_Hg_1-*y*_Te upper barrier layer were grown one by one, followed by a 50-nm CdTe cap layer (see Table 1). The structures were not intentionally doped. In all samples, there were 2D carriers in QWs at liquid helium temperature at dark conditions. Sample 1 was semimetallic, which was confirmed by transport measurements. In sample 2, the dark electron concentration was about 4×10^10^ cm^-2^ and it can be raised up to 10^11^ cm^-2^ by visible (or near-IR) light illumination (positive persistent photoconductivity (PPC)). In sample 3, the dark electron concentration was about 10^11^ cm^-2^, but, in contrast to the previous sample, visible (or near-IR) light illumination resulted to the concentration decrease down to complete freezing of free carriers (negative PPC).

**Table 1:** Sample parameters

**Sample number**	**Growth number**	**QW width (nm)**	***x***_**QW**_**(%)**	***y***_**Bar**_**(%)**	**Band structure**
1	110615	20.2	0	65	Inverted
2	100709	33	15.3	63.5	Normal
3	110621	4.8^a^	0	70	Normal

^a^Determined from PC measurements.

CR studies were carried out at *T*=4.2 K on 5×5 mm samples placed in the liquid helium. We used two superconducting coils having maximum magnetic fields of 3 and 11 T. CR spectra were measured in the Faraday configuration in two ways: by sweeping the magnetic field up to 3 T at a constant frequency of the terahertz radiation and in a static magnetic field up to 11 T. In the first case, the radiation was generated using quantum cascade lasers (QCLs) operating at 2.6, 3.2, and 4.3 THz (pulse length 10 *μ*s, repetition rate 5 to 10 kHz). The radiation transmitted through the sample was detected using a Ge:Ga impurity photodetector. In the second case, a BRUKER 113V Fourier transform (FT) spectrometer (Bruker Optik GmbH, Ettlingen, Germany) was used with a globar radiation source. The spectral resolution was 4 cm^-1^. The transmitted radiation was detected by a composite bolometer. The measured spectra presented here were normalized by sample transmission at *B*=0 and then divided by the rate of reference signals (signal without sample) at nonzero and zero magnetic fields. The latter enables us to eliminate the influence of the magnetic field on the bolometer sensitivity.

CR measurements in static magnetic fields up to 11 T were carried out in the Laboratoire National des Champs Magnétiques Intenses in Grenoble (LNCMI-G). All other measurements were performed at the Institute for Physics of Microstructures in Nizhny Novgorod.

### Theoretical calculations

The band structure in the absence of the magnetic field and the Landau levels (LLs) in the QWs under study were calculated in the axial approximation in the same way as described in [19,21] in the four-band model. The calculation is based on the envelope function method proposed by Burt [22]. The envelope functions were found as the solutions of the time-independent Schrödinger equation with the 8·8 Hamiltonian taking into account a built-in strain. To calculate the envelope functions and the corresponding values of the electron energy, the structure was approximated by a superlattice of weakly interacting QWs. The lattice period was chosen such that the interaction between the wells would not significantly affect the energy spectrum. The calculation was performed by expanding the envelope functions in plane waves. The expression for the Hamiltonian of the heterostructure grown on the (013) plane was derived by the method described in [23]. The components of the built-in strain tensor were calculated with the use of the formulas from [21]. The band parameters of the materials used in the calculation are taken from [21]. Two parameters of the model were adjusted to get better agreement between calculated and measured transition energies. The first parameter is the valence band offset of CdTe and HgTe. This parameter is not known well and, according to [23], is 570 ± 60 meV. We have used the value 620 meV. The second parameter is the Kane parameter _*EP*_which is the same for both materials according to the model used. We assumed *E*_*P*_ as 20.8 eV (instead of 18.8 eV [23]). The difference between the results of our calculations with ‘traditional’ and ‘adjusted’ parameters is shown on fan charts for all three samples under study. The dependences of all parameters, except the bandgap, on the content of the solid solution Hg_1-*x*_Cd_*x*_Te were assumed to be linear in *x*. The concentration dependence of the bandgap was described by the formula from [23]. It should be noted that the axial approximation we used is quite good for the conduction band but can give a small error for the valence band (see, for example, Figure one in [2]). According to our estimations, using axial approximation could result in the error in LL energies in the valence band up to 2 meV (16 cm^-1^).

**Figure 1:**
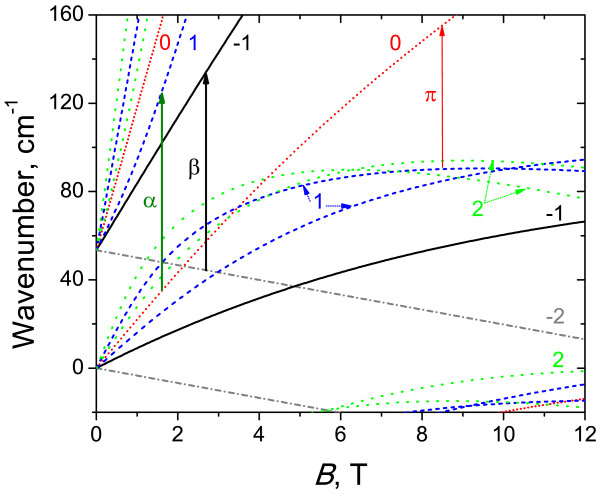
**Calculated Landau levels for sample 1 using the adjusted parameters.** Integers denote the number *n* of the Landau levels. Vertical arrows indicate the experimentally observed transitions.

Figure 1 exemplifies the fan chart of calculated LLs in sample 1. According to calculation results of the energy band spectra in the absence of the magnetic field in this sample, there is a strong overlapping between the conduction band and the side maxima in the valence band. As easy to see from Figure 1, in this sample, in addition to crossing of the lowest LL *n* = − 2 in the conduction band and the ‘top’ LL *n* = 0 in the valence band (typical for narrow QWs with inverted band structure [6,7,19]), LLs with high numbers in the valence band indeed overlap with those in the conduction band. Therefore, at the Fermi level position between 40 and 80 cm^-1^, this structure would be semimetallic with 2D electrons and holes coexisting in HgTe QW at the thermal equilibrium.

## Results and discussion

**Figure 2:**
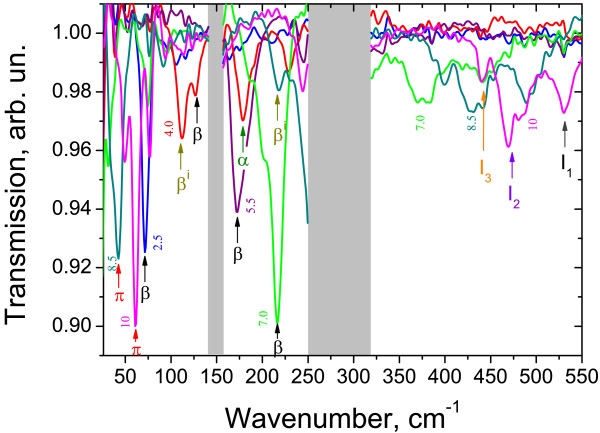
**Typical CR spectra for sample 1.** The numbers against the CR lines are the magnetic field values in Tesla. Gray stripes are Reststrahlen bands.

**Figure 3:**
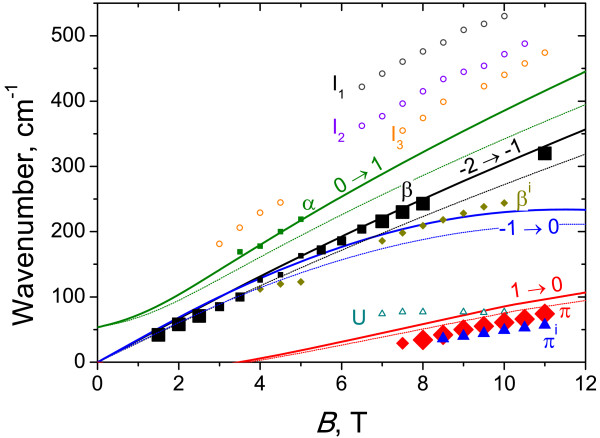
**Energies of cyclotron transitions versus the magnetic field for sample 1.** Solid lines correspond to the calculated transitions with adjusted parameters; thin dotted lines, with traditional parameters. Symbols are experimental data. The size of symbols indicates the intensity of CR lines: the smallest symbols correspond to weak lines.

Figure 2 presents typical CR spectra in sample 1 with inverted band structure obtained with a FT spectrometer. The positions of all observed absorption peaks versus the magnetic field are plotted in Figure 3. The symbol size characterizes the line intensity: bigger points correspond to more intense lines. The calculated energies of allowed transitions between LLs (Δ*n*=1) are also plotted in Figure 3. There are two stronger lines in the spectra: line *β* and line *Π*. In high magnetic fields, line *β* definitely arises from the transition between *n* = − 2 and *n* = − 1 LLs (*cf.*[6,7,19]). In this case, LL *n* = − 2 is fully occupied, level *n* = − 1 is empty (see Figure 1), and a −2→−1 transition is allowed, so we have a strong line in the spectra. In moderate magnetic fields, line *β* can also be attributed to a −1→0 transition in the conduction band: at *B*<4 T, energies of −2→−1 and −1→0 transitions are closed to each other; as the magnetic field decreases, the occupancy of LL *n* = − 1 in the conduction band in the semimetallic sample 1 increases, so the intensity of the −2→−1 transition goes down while that of the −1→0 one increases. Weak line *β*^i^, observed in high magnetic fields below line *β*, in our opinion, can be attributed to electron transitions between LL *n* = − 2 and residual donor states pertained to LL *n* = − 1.

The second strong line *Π* is a hole CR apparently. It crosses *X*-axes in a nonzero magnetic field (≈5 T), which means that the transition takes place between LLs crossing approximately in this field. The only allowed transition satisfying this condition is the 1→0 one in the valence band. Some discrepancy between measured and calculated energies (see Figure 3) is due to violation of axial approximation. Thus, line *Π* is the first observed hole CR in HgTe QWs in quantizing magnetic fields. A weaker line *Π*^i^can be, by analogy, attributed to the transitions between the filled LL *n* = 1 in the valence band and impurity state pertained to empty LL *n* = 0.

In the magnetic field range 3.5 to 5 T in the CR spectra in sample 1, we have observed a weaker line *α* that is known to result from the interband transition 0→1[6,7,19]. In *B* < 3.5 T, LL *n* = 1 seems to be occupied and the absorption decreases, while in *B*>5 T, the ‘initial’ level *n* = 0 seems to rise over the Fermi level.

Weak high-frequency lines I_1_ to I_3_ probably resulted from some interband transitions (cyclotron or impurity). At the moment, it is difficult to identify them only because of the great number of allowed transitions between valence and conduction band LLs in this frequency range. At last, the line U whose spectral position does not depend on the magnetic field most probably resulted from transitions between impurity states pertained to LLs *n* = − 2 in the valence and conduction bands (since direct transitions between these two LLs are forbidden in the Faraday configuration).

**Figure 4:**
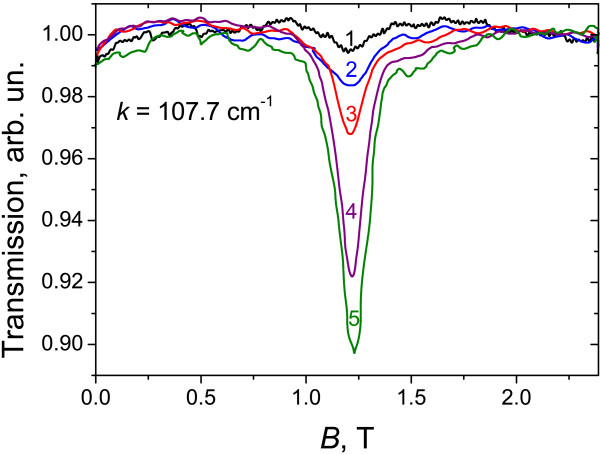
**Typical CR spectra for sample 2 measured using 3.2-THz QCL.** In the absence of visible light illumination (1) and at various levels of illumination (2 to 5). The carrier density in units of 10^10^ cm^-2^ is 3.5 (1), 5.4 (2), 7.2 (3), 9.3 (4), and 10.3 (5).

**Figure 5:**
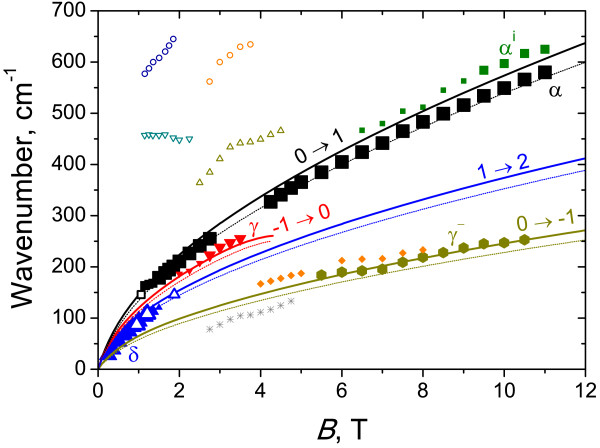
**Energies of cyclotron transitions versus the magnetic field for sample 2.** Solid lines correspond to the calculated transitions with adjusted parameters; thin dotted lines, with traditional parameters. Symbols are experimental data. The size of symbols indicates the intensity of CR lines: the smallest symbols correspond to weak lines.

Investigations of CR absorptions in sample 2 also revealed a lot of spectral features. In this sample, in addition to the magnetoabsorption study with a FT spectrometer, we also measured CR with QCLs at different 2D electron concentrations varied using the positive PPC effect. As easy to see from Figure 4, the rise of the electron concentration results in the increase of the CR line intensity only while its position is unchanged. This is an indication that the observed CR line resulted from transitions from one and the same LL (namely *n* = 1 in the conduction band; see Figure 5) because in classical magnetic fields, a gradual shift of the CR line to higher magnetic fields with the concentration increase is observed [19].

Experimental data obtained in sample 2 with both the FT spectrometer and the QCLs, as well as calculated energies of allowed transitions between conduction band LLs versus magnetic field are presented in Figure 5. It is clearly seen that the data obtained with different techniques correspond fairly well (see lower left corner in Figure 5). Besides, using QCL operating at 4.3 THz made it possible to measure CR in the phonon absorption band around 150 cm^-1^ (see Figure 2) due to a high stability of QCL radiation intensity.

The main lines in absorption spectra in sample 2 are *α*, *γ*, and *δ*. This sample has a normal band structure; therefore, all the transitions take place within the conduction band. The LL structure is analogous to that of sample 100708 studied earlier (see Figure one in [14]). Line *α* corresponds to the transition 0→1 from the lowest LL in the conduction band. In high magnetic fields over 4 T, the LL filling factor is less than unity and all the electrons in the QW occupy LL *n*=0; therefore, only CR line *α* is observed. However, in lower magnetic fields, the electrons populate the next LL *n* = − 1 (see Figure one in [14]) and the transitions −1→0 (line *γ*) are observed. At still smaller magnetic fields, the third LL in the conduction band is occupied that leads to a decrease in the intensity of transition 0→1 (line *α*) and in the appearance of line *δ*(transition 1→2).

The observed intensive absorption line *γ*^−^ is to be considered separately. Its position corresponds fairly well to the transition between two lowest LLs 0→−1. In magnetic fields over 5.5 T, where this line is observed, LL *n* = 0 is filled while that of *n* = − 1 is empty. However, according to our calculations within the axial model, the square of the electrodipole matrix element for this transition is by 4 orders of magnitude less than that for transition 0→1 (line *α*). Actually, the 0→−1 transition corresponds to electron spin resonance that should not be observed in the Faraday configuration. Nevertheless, line *γ*^−^ is clearly seen in the absorption spectra. Probably, this line resulted from transitions between shallow-donor impurity states pertained to LLs 0 and − 1. It is also possible that because of the absence of the axial symmetry in reality, the square of the matrix element for this transition will be significantly higher. In any case, the origin of line *γ*^−^ (which has been observed in a number of samples with normal band structure) requires further investigations.

Weak line *α*^i^ seems to result from the transition between the 1*s*-like state of residual shallow donors pertained to LL *n* = 0 and the excited 2*p*^ + ^-like state pertained to LL *n* = 1. In contrast to impurity lines *β*^i^ and *Π*^i^ observed in sample 1 with inverted band structure (Figure 3), the energy of the transitions corresponding to line *α*^i^ exceeds that of line *α* since the binding energy of the 1*s*-like ground state is greater than that of the excited 2*p*^+^-like state. The origin of other weak lines observed in the absorption spectra in sample 2 requires further studies.

**Figure 6:**
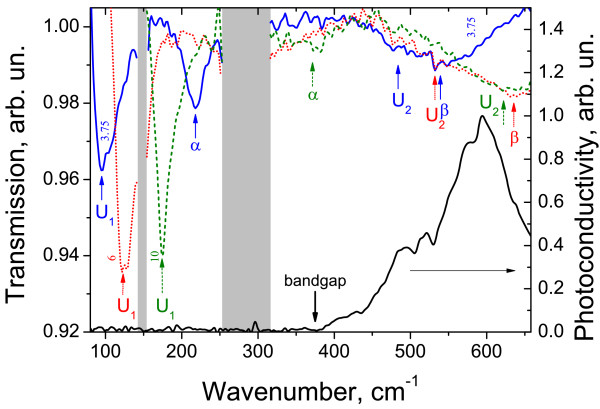
**Typical CR spectra and photoconductivity spectrum for sample 3.** The numbers against the CR lines are the magnetic field values in Tesla. Arrows indicate the observed cyclotron peaks. Gray stripes are Reststrahlen bands. The ‘bandgap’ mark indicates the value of the bandgap for 4.8-nm HgTe QW.

The last sample 3 under study contains narrow HgTe QW with nominal width of 6.3 nm that should correspond to zero bandgap [1,2]. In contrast to the previous one, this sample demonstrated negative persistent photoconductivity at illumination by visible light down to electron freezing out. The latter enables us to measure the spectrum of interband photoconductivity (Figure 6; *cf.*[13]). One can see a distinct low-frequency edge of the conductivity at 380 cm^-1^ (47 meV). According to the theoretical model used, the gap value of 47 meV corresponds to a significantly narrower QW with normal band structure. Therefore, general features of the LL fan chart in this sample are the same as those in sample 2 (see Figure one in [14]).

**Figure 7:**
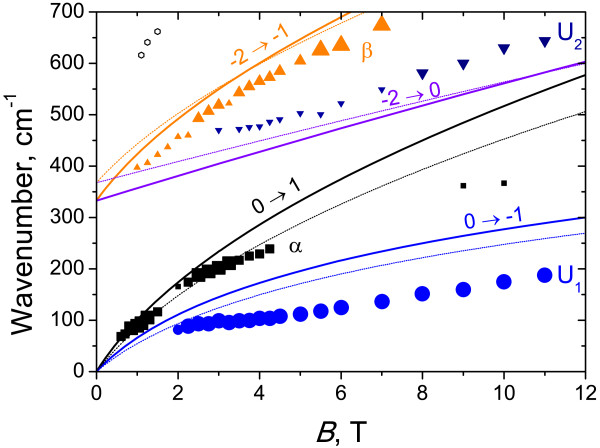
**Energies of cyclotron transitions versus the magnetic field for sample 3.** Solid lines correspond to the calculated transitions with adjusted parameters; thin dotted lines, with traditional parameters. Symbols are experimental data. The size of symbols indicates the intensity of CR lines: the smallest symbols correspond to weak lines.

Typical CR spectra in sample 3 are plotted in Figure 6, and the overall data are presented in Figure 7 together with calculated energies of CR transitions versus the magnetic field. There are four main lines in the spectra to be considered: *α*, *β*, U_1_, and U_2_. The nature of lines *α* and *β* is well known. As discussed above, line *α*corresponds to the transition from the lowest LL in the conduction band 0→1. Line *β* corresponds to the interband transition from the top LL in the valence band to the conduction band −2→−1. The intensity of this line decreases in magnetic fields below 3 T (see Figure 7) because of partial filling of the ‘final’ LL for this transition *n* = − 1. To the best of our knowledge, this is the first observation on the interband transition in the HgTe QW with normal band structure. At present, such transitions have been observed in HgTe QWs with inverted band structure only (see, e.g., [6,7,19]). It should be mentioned that the extrapolation of the spectral position of line *β* to *B* = 0 gives slightly less bandgap (340 cm^-1^) than that obtained from the photoconductivity spectrum (measured on another sample cut from the same wafer). Therefore, in our calculations, we used a compromised (between CR and photoconductivity data) QW width of 4.8 nm. Let us note that in this sample 3 with the narrowest QW, the linewidth of the interband transition (*β*) exceeds significantly that of the intraband one (*α*), while in broad QWs, they are approximately the same (see, e.g., Figure 2; [7,19]). To our opinion, a spreading of the interband line *β* in sample 3 with narrow QW resulted from the enhanced role of one-monolayer fluctuations (about 0.5 nm) of this narrow QW width which, in turn, leads to bandgap fluctuations.

The nature of the most intense low-frequency line in CR spectra U_1_ is not quite clear. It persists up to the maximal magnetic field used (11 T) when the LL filling factor is much less than unity, so it cannot be attributed to transitions between higher LLs in the conduction band. On the other hand, the transition energies are much less than the bandgap. Therefore, the only reasonable explanation is to attribute this absorption line to intracenter excitation of residual donors. In wide QWs (such as in sample 2), the shallow-donor binding energies are small compared to those of the CR ones because of small electron effective masses (of the order 10^-2^*m*_0_, where *m*_0_ is the free electron mass). However, in narrow QWs, the donor binding energies increase significantly since the QW potential pushes the donor wavefunction to the impurity ion. A weaker absorption line U_2_ seems to result from some impurity interband transition since the line is as broad as line *β*. As a whole, the accordance between measured and calculated data in sample 3 with the narrowest QW (Figure 7) is worst than those in samples 1 and 2 with wider QWs. The latter means that the theoretical model for the description of such narrow QWs is to be elaborated.

## Conclusions

In conclusion, we have measured CR in a set of nominally undoped HgCdTe QWs with different band structures in quantizing magnetic fields. The results obtained are interpreted on the basis of Landau level calculations within the Kane 8·8 model. In wide semimetallic HgTe QWs with inverted band structure, both intra- and interband transitions between Landau levels are identified, the CR line being accompanied by impurity satellites. A hole CR line has been observed for the first time. In two samples with normal band structure: wide (30 nm) HgCdTe QW and narrow (4.8 nm) HgTe QW, interband CR transitions have been revealed in the spectra, the interband absorption line width in the narrow QW being spread due to QW width fluctuations. The adjusted material parameters: valence band offset of CdTe and HgTe 620 meV (instead of 570 meV) and the Kane parameter *E*_*P*_20.8 eV (instead of 18.8 eV), are proposed from the comparison of the experimental and calculation data.

## Abbreviations

2D: two-dimensional; CR: cyclotron resonance; IR: infrared; LL: Landau level; PC: photoconductivity; PPC: persistent photoconductivity; QCL: quantum cascade laser; QW: quantum well.

## Competing interests

The authors declare that they have no competing interests.

## Authors’ contributions

MSZ, FT, and MO performed the CR measurements using the Fourier transform spectrometer. MSZ carried out all calculations. AVI and KVM performed the CR measurements with QCL. AVI performed the photoconductivity measurements. KES characterized the samples with magnetotransport measurements. SAD and NNM grew the experimental samples with MBE technology. AVI, VIG, WK, and MSZ explained the obtained data. AVI and VIG wrote the manuscript draft. All authors read and approved the final manuscript.
